# RNA-seq as a powerful tool for penaeid shrimp genetic progress

**DOI:** 10.3389/fgene.2014.00298

**Published:** 2014-08-28

**Authors:** Camilla A. Santos, Danielly V. Blanck, Patrícia D. de Freitas

**Affiliations:** Laboratory of Molecular Biodiversity and Conservation, Department of Genetics and Evolution, Federal University of São CarlosSão Carlos, Brazil

**Keywords:** differential expression analysis, differential phenotype, high-throughput transcriptome, penaeidae transcriptome, candidate genes

## Abstract

The sequences of all different RNA transcripts present in a cell or tissue that are related to the gene expression and its functional control represent what it is called a transcriptome. The transcripts vary between cells, tissues, ontogenetic and environmental conditions, and the knowledge that can be gained through them is of a solid relevance for genetic applications in aquaculture. Some of the techniques used in transcriptome studies, such as microarrays, are being replaced for next-generation sequencing approaches. RNA-seq emerges as a new possibility for the transcriptome complexity analysis as well as for the candidate genes and polymorphisms identification of penaeid species. Thus, it may also help to understand the determination of complex traits mechanisms and genetic improvement of stocks. In this review, it is first introduced an overview of transcriptome analysis by RNA-seq, followed by a discussion of how this approach may be applied in genetic progress within penaeid stocks.

## INTRODUCTION

The term RNA-seq has been used to make reference to a transcriptome produced by methods of next generation sequencing (NGS), which ensure a good coverage of transcripts detection, due to the sequencing of millions of reads ranging from 25 to 300 bp, depending on the platform used (Wang et al., 2009; Oshlack et al., 2010). The full set of transcripts in a cell is known as transcriptome. They involve all types of ribonucleic acids (RNAs), including the protein coding messenger ribonucleic acid (mRNA) and the non-coding ribonucleic acid (ncRNA) such as ribosomal RNAs (rRNA), transfer RNAs (tRNA), and the small nuclear RNAs (snRNA). These RNAs may be differentially expressed according to the tissue, the stage of development and the physiological condition being accessed (Wang et al., 2009; Anders and Huber, 2010).

Transcriptome studies have been widely conducted in order to identify new genes, prospect simple sequence repeats (SSR) and single nucleotide polymorphisms (SNP) markers and to analyze differentially expressed genes. Such approaches have been helping to understand different mechanisms related to cellular control and describe important metabolic pathways, what enables a better understanding of the genotype–phenotype relationship (Marguerat and Bähler, 2010; Khatri et al., 2012; Qian et al., 2014).

Small and large scale transcriptome analyses and differential expression studies, such as Expressed Sequence Tags (ESTs) and microarrays, have been carried out in some penaeid shrimp species (Rojtinnakorn et al., 2002; La Vega et al., 2007; James et al., 2010; Brady et al., 2013). However, RNA-seq approaches are still incipient in shrimp (**Table 1**; Li et al., 2012, 2013; Guo et al., 2013; Sookruksawong et al., 2013; Xue et al., 2013; Zeng et al., 2013; Baranski et al., 2014; Yu et al., 2014). Therefore, the method herein has emerged as a new possibility for the transcriptome complexity analyses in face of varied production and/or experimental conditions. Consequently, such approach aims to develop genetically improved strains, focusing mainly on the resistance factor.

**Table 1:** Summary of studies on penaeid shrimp using the RNA-seq approach.

Reference/species	NGS platform	Application	Annotation of genomic information
Baranski et al. (2014)/*Penaeus monodon*	Illumina	SNPs and microsatellite prospecting	136,223 contigs; 473,620 SNPs
Li et al. (2013)/*Fenneropenaeus chinensis*	Illumina	Immune response to WSSV	46,676 unigenes; 805 differentially expressed genes
Yu et al. (2014)/*Litopenaeus vannamei*	Illumina	SNPs prospecting	96,040 SNPs
Guo et al. (2013)/*L. vannamei*	Illumina	Stress; immune response; apoptosis	42,336 unigenes; 2,445 differentially expressed genes
Sookruksawong et al. (2013)/*L. vannamei*	Illumina	Immune response to TSV	61,937 contigs; 1,374 differentially expressed contigs
Zeng et al. (2013)/*L. vannamei*	454	Immune response to TSV; microsatellites prospecting	15,004 unigenes; 770 microsatellites; 1,311 differentially expressed genes
Xue et al. (2013)/*L. vannamei*	Illumina	Immune response to WSSV	52,073 unigenes; 1,179 differentially expressed genes
Li et al. (2012)/*L. vannamei*	Illumina	Genomic annotation	109,169 unigenes

In order to obtain a transcriptome via RNA-seq, some steps should be followed: (i) selection of tissue of interest and isolation of RNA molecules; (ii) construction of cDNA libraries; (iii) utilization of a NGS platform; and (iv) the reads analysis in order to establish unigenes and the transcriptome assemble through bioinformatics tools.

The tissue choice should be based on the study aim and/or the genes nature to be analyzed. As a parallel, a transcriptome consists in taking a photograph from a specific time in a cell, highlighting only the condition at that short period of time. In this manner, tissue selection and the suitable time to perform a transcriptome requires preparation, otherwise the experiment as a whole may be biased (Wang et al., 2009). Libraries establishment is crucial for the final result in face of the many laboratorial procedures that are conducted, leading to some biases in the obtained results (Wang et al., 2009). Bioinformatics analysis is also an important step and includes the use of computational tools that guarantee the processing of large volumes of data generated by next-gen (Gavery and Roberts, 2012; Guo et al., 2013).

Within this review, it is presented a brief overview of the RNA-seq method, including its main advantages and limitations. Following that, it will be discussed how such technique may be applied to obtain genetic progress in penaeid shrimp farming.

## RNA-seq: ADVANTAGES AND LIMITATIONS

The transcriptome assembly may be based on a reference genome available (Wang et al., 2009), which allows to quickly locate similar regions using local alignment algorithms and presents higher reliability due to the large volume of small sized reads coming from alternative splicing. It equally provides a more even coverage of the genome (Anders and Huber, 2010; Qian et al., 2014). On the other hand, even when there is no reference genome available, *de novo* transcriptome assembly may be carried out using specific algorithms, which stands as a solid advantage for some species that have not been widely studied yet (Howe et al., 2013; O’Neil and Emrich, 2013).

When performing transcriptome via RNA-seq, a high coverage is obtained, which allows the discovery of new genes and polymorphisms (Marguerat and Bähler, 2010; Yu et al., 2014). Li et al. (2012) evaluated the abundance and coverage of transcriptomes obtained by RNA-seq in *Litopenaeus vannamei*. By comparing such data to the ESTs available on GenBank, it was found that only 14.2% (15,519 out of 109,169) of unigenes obtained by RNA-seq were also found in the EST libraries, generating a lot of new informative data. In addition to that, the wide coverage associated with high resolution provided by this technique ensured high accuracy in SNP discovery in coding genes (Yu et al., 2014).

That taken, RNA-seq allows the detection of variations in a single nucleotide, enabling the detection of the expression of protein isoforms and their respective allelic variants, characterizing SNPs (Baranski et al., 2014; Yu et al., 2014). Polymorphic microsatellites or SSRs have equally been identified through RNA-seq analysis (Mohd-Shamsudin et al., 2013; Zeng et al., 2013; Baranski et al., 2014). On those cases, though, a wider coverage of the reference genome is suggested (Qian et al., 2014) once the presence of highly repetitive regions could stand as a limiting factor by compromising the transcriptome assembly.

As could be observed, RNA-seq has been considered a solid method for the large-scale gene expression analysis due to the fact it does not require prior genome knowledge (Wang et al., 2009) and enables the detection of isoforms arising from alternative splicing (Ghosh and Qin, 2010). Even when involving several samples, such technique is accessible with moderate costs. In this case multiplex runs containing up to 10 samples per sequencing lane can be performed in some platforms and the costs are no longer a limiting factor.

Another RNA-seq advantage is its wide dynamic range (ratio between the minimum and maximum expression level). This feature makes it suitable for measuring low, medium and high expression levels of the genes, not requiring very sophisticated normalization. By contrast, DNA microarrays show reliable results only for medium expression levels and therefore have a much smaller dynamic range. Thus, RNA-seq provides much more informative data, requiring less biological material and lower costs, becoming this technique popular for measuring gene expression on a large scale (Sharov et al., 2004; Wang et al., 2009).

## RNA-seq APPLICATION WITHIN PENAEID SHRIMP AQUACULTURE

The use of RNA-seq in species of penaeid shrimp can be focused on transcriptome characterization, functional annotation, gene expression profiles analysis, and gene-associated markers identification. In this section, the emphasis is given to the analysis of differential expression, identification of molecular markers, and its potential to promote genetic gain and development of improved penaeid strains. Similar studies have allowed the identification of candidate genes or quantitative trait loci (QTLs), which could be related to traits of interest for aquaculture, such as reproduction, sex determination, growth, immunity, and tolerance against environmental stress. Data involving pathways are also relevant in order to obtain more details about the interaction mechanism between the expressed products and their importance and applicability.

### IDENTIFYING CANDIDATE GENES THROUGH DIFFERENTIAL EXPRESSION ANALYSIS

Although the applicability of RNA-seq in transcriptome and differential expression in aquatic organisms have increased in the past 3 years, the results found in literature and in the Sequence Read Archive Database of the National Center for Biotechnology Information (SRA-NCBI) indicate that such approach still is incipient for penaeid shrimp. SRA databank, per instance, presents only 28 deposits of data generated by NGS in what regards the species *L. vannamei*, *L. stylirostris,* and *Penaeus monodon* (). In literature, it has been found next-gen data only for *P. monodon* (Baranski et al., 2014) *Fenneropenaeus chinensis* (Li et al., 2013) and *L. vannamei* (Li et al., 2012; Guo et al., 2013; Sookruksawong et al., 2013; Xue et al., 2013; Zeng et al., 2013; Yu et al., 2014). Mostly, the research in this field has been covering the identification of genes connected to immunity, mainly concerning the white spot syndrome virus (WSSV) and the taura syndrome virus (TSV; Li et al., 2013; Sookruksawong et al., 2013; Xue et al., 2013; Zeng et al., 2013; Baranski et al., 2014). Both syndromes have caused great economic losses for the shrimp industry throughout the past few decades.

**FIGURE 1:**
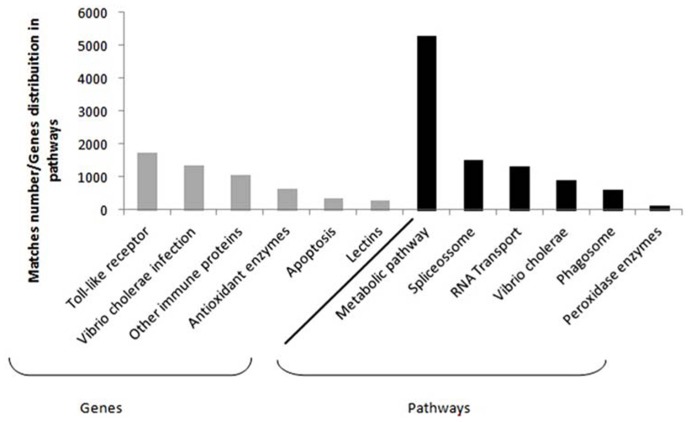
**Compilation based on the immune genes in Supplementary Material available online from seven studies.** Details from the study and the respective files are as follow: Li et al. (2012; Tables S5, S6 and S7), Li et al. (2013; Tables S3 and S6), Sookruksawong et al. (2013; Tables S5 and S6), Zeng et al. (2013; Tables S4 and S5), Xue et al. (2013; Table S1), Baranski et al. (2014; Tables S5 and S6), and Yu et al. (2014; File 1). The most frequent genes found are in gray and the main pathways in which these genes are distributed can be seen in black.

Despite the fact that crustaceans do not own an immune system, some candidate genes have been obtained from hemolymph and hepatopancreas tissues. It is clearly seen in some differential expression studies concerning *L. vannamei* species, which represents the biggest portion of the marine shrimp worldwide production (Gucic et al., 2013). Among the main genes studied, it is possible to find those related to toll-like and signalizing receptors, apoptosis, *Vibrio cholerae* infection and other immune proteins (e.g., phagosome, hemocyanin, crustacyanin, antiviral), antioxidant enzymes (the peroxidases and glutathione ones), and lectins (**Figure 1**; Li et al., 2012, 2013; Sookruksawong et al., 2013; Xue et al., 2013; Zeng et al., 2013; Baranski et al., 2014; Yu et al., 2014).

Data related to toll-like and lectin proteins demonstrate that those may act as signaling molecules, what causes the increase of peptides expression responsible for controlling the immune response (Wang et al., 2014). On the other hand, genes associated with apoptosis may indicate an attempt to prevent proliferation of viruses and possible damages to genetic material, through death of infected cells. Specifically in what regards large number of proteins related to infection by *V. cholerae* response is due to the recurring presence of this group of bacteria within shrimp farming tanks (Banerjee et al., 2012).

Information regarding the main metabolic pathways and the quantity of most frequent genes in each pathway were also collected, as part of the data obtained via functional annotation for RNA-seq. In penaeid, the most commonly described pathways were those involving the general metabolism, spliceosome, RNA transport, *V. cholerae* infection, phagosome and the antioxidant ones, which include peroxidase enzymes (Li et al., 2012, 2013; Sookruksawong et al., 2013; Xue et al., 2013; Zeng et al., 2013; Yu et al., 2014). Spliceosome and RNA transport pathway supposedly act in new transcripts formation, providing genetic variants that may contribute to resistance (Yang et al., 2007).

Regarding Gene Ontology (GO) categories, all studies in penaeid have mainly reported the same data. Considering the biological processes, per instance, the most frequent were metabolism and biological regulation. In what regards cellular components, genes are mostly expressed at the cell and some unspecific organelles. Finally, concerning the molecular function, the most common ones were catabolic activity and binding (Li et al., 2012, 2013; Sookruksawong et al., 2013; Xue et al., 2013; Zeng et al., 2013; Baranski et al., 2014; Yu et al., 2014). Overall results such as these were expected, since the penaeid species previously mentioned herein have too little information about their genomes available. In the case of *L. vannamei*, only approximately 12,000 gene products were described, which may be useful in a comparative approach concerning a *de novo* assembly ().

### IDENTIFYING GENE ASSOCIATED MARKERS

In what regards RNA-seq technology, it has also been proving to be an extremely useful tool for identifying SNPs, which may be also used to develop high density SNPs chips for studies concerning the genome wide association (GWAS) and to build high density linkage maps (Baranski et al., 2014; Yu et al., 2014). Furthermore, SNPs can be used as markers in order to distinguish allelic transcripts whilst studying the allele-specific expression (Bell and Beck, 2009).

In a recent study, Yu et al. (2014) prospected SNPs in *L. vannamei*. A total of 58,717 unigenes and 36,277 high quality SNPs were predicted by transcriptomes “*M”* (produced by the authors themselves) and *“P”* (downloaded from SRA database, session number SRR346404, which was published by Li et al., 2012), respectively. Those SNPs were spread out among 25,071 unigenes and allocated to 254 pathways at the KEGG (Kyoto Encyclopedia of Genes and Genomes) database. The main pathways containing high number of SNPs were metabolic pathways, amoebiasis, *V. clolerae* infection, RNA transport, and actin cytoskeleton regulation.

Baranski et al. (2014) used the approach to build a high density linkage map in *P. monodon*. A total of 6,000 out of 473,620 SNPs/indels putative were genotyped by using the Illumina iSelectCerca genotyping matrix. Out of those SNPs, 3,959 were mapped in 44 linking groups and out of those 2,340 were functionally annotated according to the GO database (see dataset S5 and S6 from Baranski et al., 2014). According to the authors, these polymorphisms may be causal or closely related to other mutations that affect important traits, such as resistance to diseases and reproductive performance.

The identification and functional annotation of SNPs identified by Baranski et al. (2014) and Yu et al. (2014) studies represent a useful resource to comprehend mechanisms determining complex traits and, consequently, to develop programs aiming the genetic improvement of these characters in penaeid shrimp strains. That taken, those SNPs can be applied both in marker assisted selection (MAS), using SNPs closely associated with QTL, and in genomic selection, through complete set of identified SNPs. That increases the rate of genetic gain per generation in traits of great interest to the shrimp industry, such as growth and resistance to disease.

## FINAL CONSIDERATIONS

One of the possible challenges that arise within genetic gain is the development of penaeid strains that may simultaneously present high growth development and pathogens resistance. Genetic correlation studies have shown that there is a negative phenotypic correlation between the resistance to diseases and the weight gained by the animals (Argue et al., 2002; Gitterle et al., 2005; Cock et al., 2009). Cock et al. (2009), reinforce the fact specimen potentially resistant to WSSV also present low reproduction efficiency. Such remarks suggest that genes with pleiotropic effects may be responsible for the trade-off observed between these traits in penaeid shrimp. From this perspective, the RNA-seq technique can be used for discovering such genes, since the overlapping of differentially expressed genes in both strains resistant to pathogens and in large weight gain strains can also be verified. Therefore, up-regulated or down-regulated genes expressed in these two strains could indicate a possible pleiotropic effect. Besides, mRNA studies allied with RNA-seq method could also be used for micro RNAs (miRNA) analyses. This approach was applied in aquaculture species, such as freshwater prawn* Macrobrachium rosenbergii* (Tan et al., 2013), and tilapia (Huang et al., 2012). As a result, it has been shown that miRNAs are critical regulators of generalized cellular functions such as differentiation, proliferation, and cell growth.

Another challenge within aquaculture is the difficulty in achieving sexual maturity and spawning of penaeid species (except for *L. vannamei*), under the farming conditions (Lo et al., 2007; Brady et al., 2013). As an attempt to overcome such problem, ablation of the eyestalk has been conducted for many years. Nevertheless, such practice is associated to high mortality rates, and low spawning and survival rates (Huberman, 2000). Considering this, the transcriptome analysis obtained from reproductive organs of native and captive specimens of penaeid shrimp via RNA-seq may significantly contribute to the identification of the underlying causes of reproductive dysfunction observed in farmed animals. Furthermore, the discovery of genes involved in gonadal maturation and reproductive performance may assist in gametogenesis, handling studies involving these species.

Finally, transcriptome and differential expression analysis by RNA-seq may be a powerful approach to optimize the penaeid diet composition (nutrigenomics), especially for those species that do not count on a specific availability of diet. The approach may be used to identify specific changes in molecular level (Chávez-Calvillo et al., 2010), which in turn also cause metabolic and physiological changes in shrimp treated with different diets (e.g., levels of crude protein, levels of plant protein inclusion and of antioxidants, vitamins, and polyunsaturated fatty acids). Thus, nutrigenomics can be used to produce healthy animals and safe and high quality products for the consumer, emerging as a promising area of research for sustainability and profitability in aquaculture (Cerdà and Manchado, 2013).

Although NGS technologies are showing their efficiency in works related to gene expression, other methodologies such as third-generation sequencing, also referred to as single-molecule sequencing (Single-Molecule Real-Time, SMRT), are being developed, but already showing limitations. More advanced techniques of sequencing are also on the way, such as “next-next-generation,” which it is capable of handling millions of DNA molecules simultaneously, including cDNAs from the RNAs.

Considering the many technologies that are already available or emerging, researchers can only venture in this world of possible and promising technologies. Various research groups should seek to unite efforts in order to overcome the difficult and challenging task of applying the enormous potential of these new methods to advance and progress in penaeid shrimp aquaculture.

## Conflict of Interest Statement

The authors declare that the research was conducted in the absence of any commercial or financial relationships that could be construed as a potential conflict of interest.
